# Improving mapping for Ebola response through mobilising a local community with self-owned smartphones: Tonkolili District, Sierra Leone, January 2015

**DOI:** 10.1371/journal.pone.0189959

**Published:** 2018-01-03

**Authors:** Laura M. Nic Lochlainn, Ivan Gayton, Georgios Theocharopoulos, Robin Edwards, Kostas Danis, Ronald Kremer, Karline Kleijer, Sumaila M. Tejan, Mohamed Sankoh, Augustin Jimissa, Jane Greig, Grazia Caleo

**Affiliations:** 1 European Programme for Intervention Epidemiology Training (EPIET), European Centre for Disease Prevention and Control, Stockholm, Sweden; 2 National Institute for Public Health and the Environment (RIVM), Centre for Infectious Disease Control, Bilthoven, the Netherlands; 3 Médecins Sans Frontières, Magburaka, Sierra Leone; 4 Manson Unit, Médecins Sans Frontières, London, United Kingdom; 5 Santé Public France, French National Public Health Agency, Saint-Maurice, France; 6 Operational Centre Amsterdam, Médecins Sans Frontières, Amsterdam, the Netherlands; 7 Tonkolili District Health Management Team, Sierra Leone Ministry of Health and Sanitation, Magburaka, Sierra Leone; Cairo University, EGYPT

## Abstract

**Background:**

During the 2014–16 Ebola virus disease (EVD) outbreak, the Magburaka Ebola Management Centre (EMC) operated by Médecins Sans Frontières (MSF) in Tonkolili District, Sierra Leone, identified that available district maps lacked up-to-date village information to facilitate timely implementation of EVD control strategies. In January 2015, we undertook a survey in chiefdoms within the MSF EMC catchment area to collect mapping and village data. We explore the feasibility and cost to mobilise a local community for this survey, describe validation against existing mapping sources and use of the data to prioritise areas for interventions, and lessons learned.

**Methods:**

We recruited local people with self-owned Android smartphones installed with open-source survey software (OpenDataKit (ODK)) and open-source navigation software (OpenStreetMap Automated Navigation Directions (OsmAnd)). Surveyors were paired with local motorbike drivers to travel to eligible villages. The collected mapping data were validated by checking for duplication and comparing the village names against a pre-existing village name and location list using a geographic distance and text string-matching algorithm.

**Results:**

The survey teams gained sufficient familiarity with the ODK and OsmAnd software within 1–2 hours. Nine chiefdoms in Tonkolili District and three in Bombali District were surveyed within two weeks. Following de-duplication, the surveyors collected data from 891 villages with an estimated 127,021 households. The overall survey cost was €3,395; €3.80 per village surveyed. The MSF GIS team (MSF-OCG) created improved maps for the MSF Magburaka EMC team which were used to support surveillance, investigation of suspect EVD cases, hygiene-kit distribution and EVD survivor support. We shared the mapping data with OpenStreetMap, the local Ministry of Health and Sanitation and Sierra Leone District and National Ebola Response Centres.

**Conclusions:**

Involving local community and using accessible technology allowed rapid implementation, at moderate cost, of a survey to collect geographic and essential village information, and creation of updated maps. These methods could be used for future emergencies to facilitate response.

## Introduction

The Ebola virus disease (EVD) outbreak in West Africa began in December 2013 in Guinea and spread to neighbouring Liberia and Sierra Leone in 2014 [1]. Between May 2014 and March 2016, Sierra Leone reported 14,124 EVD cases and 3,956 EVD-related deaths [2], making it one of the countries most affected by an EVD outbreak.

Tonkolili District is one of twelve districts in Sierra Leone, situated in the Northern Province of the country. The district is 7,003 km2 and administratively divided into 11 chiefdoms, with Magburaka as its capital and largest city. Magburaka also serves a number of chiefdoms from Bombali District, which borders Tonkolili District. The 2004 Sierra Leone Population and Housing Census, the most recent census available at the time of the outbreak, reported Tonkolili District having a population of 347,197 inhabitants, and the two chiefdoms near Magburaka in Bombali District a population of 39,693 inhabitants [3].

Between May 2014 and April 2016, Tonkolili District reported 406 EVD cases including 162 EVD deaths [MSF, personal communication]. Tonkolili District did not have an Ebola Management Centre (EMC) until December 2014, when Médecins Sans Frontières (MSF) constructed an EMC in Magburaka. The EMC was constructed in 12 days, with a 100-bed capacity, medical and hygiene team, an on-site laboratory, epidemiology, water and sanitation, health promotion, outreach and mental health counselling teams.

With each new admission to the Magburaka EMC, MSF staff recorded all patient information, including the origin of the patient, through patient interview or from the ambulance team if the patient was unconscious. This information was entered into a line list by MSF data encoders based in the Magburaka EMC. Information regarding EVD positive cases was shared with the local Ministry of Health and Sanitation (MoHS) and Ebola response partners. However, MSF staff and partners were often confronted with challenges in obtaining reliable geographical information to enable the immediate tracing of EVD contacts and follow-up of discharged survivors. This was because the village of origin given by the patient could sometimes not be located on the available district maps, as some villages had similar names but were in different chiefdoms, or villages had both an official and an alternate village name. In addition, information about new villages, including satellite villages and up-to-date population numbers, were not available. This delayed the response, and MSF and MoHS staff often relied on knowledge of their local drivers, or had to stop and ask local residents for village locations.

Timely and effective containment of EVD relies on multiple interventions: isolation, surveillance, contact tracing, decontamination, health promotion, psychosocial support and community engagement [4]. These interventions cannot be implemented without accurately locating potentially affected areas. Therefore, accurate maps are an important tool for outbreak investigation and response, and facilitate visualising the extent of an outbreak [5].

In order to improve EVD response, we describe a survey conducted in January 2015 to explore the feasibility and cost to mobilise a local community with their own smartphones in the collection of vital mapping and village information from chiefdoms within the MSF EMC catchment area in Tonkolili and Bombali Districts. We also describe the process developed to validate the mapping data, the use of this data to prioritise areas for interventions, and lessons learned.

## Methods

### Mobilisation of local people

We recruited survey teams in Magburaka town by selecting local residents as surveyors based on their ownership of a fully functional Android smartphone, fluency in both Temne and Mende (local languages) and a good command of English. To transport the surveyors, we selected local commercial motorbike drivers known as ‘Okada’ drivers. Okada drivers are a popular mode of transport in Sierra Leone due to their affordability, in-depth knowledge of the villages and ability to navigate the sometimes difficult terrain in remote areas of Tonkolili District and neighbouring chiefdoms in Bombali District.

One surveyor was designated as survey team leader based on their natural leadership of the team during training, rapid acquaintance with the technology and knowledge of the areas to be surveyed in Tonkolili and Bombali chiefdoms. Their role was to oversee tasks and areas for the survey teams to cover. Each survey team, comprising of one Okada driver and one surveyor, were initially sent to villages close to the MSF Magburaka EMC. The teams were then dispersed to chiefdoms within the catchment area of the MSF Magburaka EMC in Tonkolili and Bombali District. The teams were unable to enter the chiefdom of Sambaya in Tonkolili District, as the paramount chief had issued a lockdown due to fear of EVD transmission in January 2015. The teams also did not visit the chiefdom of Kunike in Tonkolili District, as there were no Ebola cases or people sought care in neighbouring districts. The surveyors and Okada drivers were also briefed by the MSF water and sanitation team on safety measures in the event of visiting Ebola hotspots.

### Training and technology sources used

We used smartphones with Android, rather than Symbian or other operating systems, due to their reasonably high market penetration and the availability of high-quality Android-compatible open-source software. The Android smartphones of the survey team members were installed with open-source software for data collection (OpenDataKit (ODK) www.opendatakit.org) and navigation (OpenStreetMap Automated Navigation Directions (OsmAnd) www.OsmAnd.net). Both are freely available for download from the Google Play store, but could also have been installed on Android smartphones via USB.

The survey teams were trained how to collect the survey data using ODK and the village location coordinates using OsmAnd. As all Android smartphones were self-owned, no training was required for device use.

### Open Data Kit (ODK) collect software and data collection

ODK is an open-source tool to create and manage mobile data collection. To maintain the security of collected data, our methodology involved some advanced setup, including changing the ODK default settings from connecting to a public website, to instead, routing the connection to a local password protected web-based aggregation platform operated by MSF.

To collect information, we designed a structured survey form with questions, prompts and cascading functions using an XLSForm, which is a form standard that simplifies authoring and modification of forms for ODK in Microsoft Excel (http://xlsform.org/). The XLSForm was then converted to an XForm, which is an open form standard supported by ODK and other data collection platforms. The finished survey form was uploaded to the MSF web-based aggregation platform, from where only authorised Android smartphones could download it (S1 Fig). A how-to guide for use of open-source software to collect mapping and survey data using smartphones can be found in S1 Text.

The survey form automatically captured information about the survey start and end time, assigned a device identification number for each Android device used, an unique identifier for each form filled and finally the time the survey was uploaded to the server. Upon manual prompt, the survey captured village geolocation (S2 Fig). The surveyors then interviewed the village chief or head of village and manually entered the following information: village name (and common variants); district, chiefdom, ward, constituency and zone of village; name and contact information of village chief and local health-care worker (HCW); approximate number of households in the village as proxy of population estimate.

Mid-way through the data collection process, we became aware that additional information would be valuable to the outbreak response. The survey form was revised in order to add: administrative section and zone; total village population (as given by the village leader); location and type of health centre in the village (or nearest health centre if none within the village); presence of a village school.

### Open Street Map Automated Navigation Directions (OsmAnd)

OsmAnd is both an online and offline mobile maps and navigation application with access to high-quality OpenStreetMap (OSM) data (S2 Fig). Prior to commencing this survey, OSM obtained old Sierra Leonean maps and digitised them. This enabled downloading of Tonkolili and Bombali District vector maps in OsmAnd.

### Ethics statement

This study was carried out as part of an operational outbreak response to enable improved and targeted health response during the Ebola outbreak. The study was approved as not requiring formal review from the MSF Ethics Review Board (ERB) by the Medical Director of MSF Operational Centre Amsterdam (MSF-OCA), as it did not involve clinical data or human subjects research [6, 7]. However, it was recommended that usual standards of care surrounding consent, data protection, and confidentiality, and harm/benefit ratio were adhered to.

Consent was obtained by verbal permission from each village head. Personal data collected during this study, such as name and telephone numbers, were not collected to be analysed as part of the research, but rather for administrative reasons. Data confidentiality and security were maintained by storing the collected data on a password protected web-based aggregation platform operated by MSF. This study was retrospectively reviewed by MSF and two ethicists during development of a self-guided ethics framework for innovation (which can include novel operational responses) projects that do not involve human subjects [8]. The review concluded that the likely benefits of this innovation significantly outweighed potential harms.

### Validation of mapping data

We calculated proportions using the non-missing values of each variable as denominator. We performed the descriptive analysis of the collected survey data using Stata 14.2 software [9].

**Fig 1:**
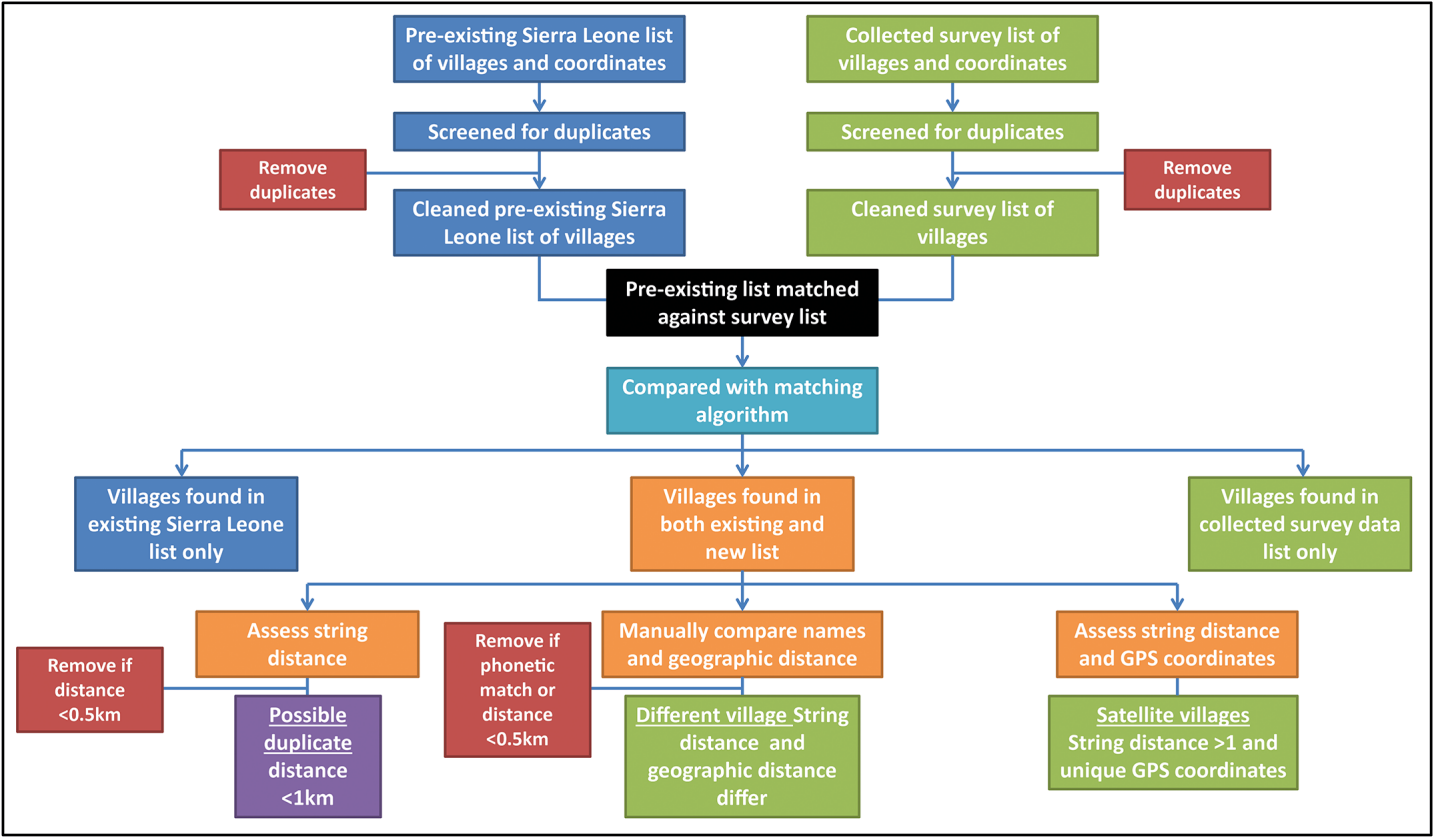
Process of validation of mapping data.

For initial validation of the mapping data, Okada drivers and Tonkolili District Health Management Team members were involved during data collection, as they have in-depth knowledge of village names in the districts. The second validation step was carried out using an algorithm. We focused our analysis on the chiefdoms extensively visited by the survey teams. The list of villages collected (survey list) was compared to a pre-existing 2004 Statistics Sierra Leone list. This was done using a matching algorithm developed internally in the open-source statistical analysis program R [10], which produced the top three possible matches between two lists of villages according to a weighted combination of the results of both geographical distance between the GPS coordinates and the string distance between the two village names (Fig 1). The Damerau–Levenshtein restricted method was used to calculate string distance—the minimum number of letter edits (additions, substitutions and/or removals) to change one word into the other. The combined metric for selecting the three possible matches accounted for differing length of village names by first normalising the string distance to the primary name length, then normalised geographic distance by scaling within the match set, and finally created a composite measure of the two normalised distances. As the same village names are known to be used in multiple chiefdoms, we compared the string distance between village names after first matching district and chiefdom.

Each list of villages (survey and pre-existing list) was first compared internally to itself and existing duplicates removed. The cleaned survey list was then matched against the cleaned pre-existing list. A pair of villages in the list were considered likely to be duplicates and assessed further for string distance if the geographic distance was less than 0.5km, and possible duplicates if it was less than 1.0km. If the location was precisely the same, the name was manually compared regardless of string distance, and assumed a duplicate if it was a phonetic match. For distances >0km and <0.5km, names were manually compared if the string distance was 0–2 letters, and considered a duplicate if it was a phonetic match. If the string distance was ≥3 letters but the geographic distance was <0.1km and it was the closest possible match, names were manually compared for phonetic similarity. If the name matched to a string distance of 1 letter and had a coordinate number at the end that differed, these were considered satellite villages, not duplicates. The number of villages that occurred in the matched list was calculated.

### Estimation of population size in surveyed chiefdoms

To calculate the estimated population size per chiefdom surveyed, the surveyors collected the number of households per village according to village chiefs’ estimates. We then applied a value of 5.9, which is the average number of people per Sierra Leonean household [11], to the number of households per village.

### Maps

We created maps using QGIS^®^ (www.qgis.org). Locations of villages were mapped from coordinates recorded with the ODK and OsmAnd software.

### Project costs

Costs were calculated considering the fixed daily cost of Okada drivers, which included fuel and vehicle maintenance costs. The daily cost for surveyors was calculated based on a fixed daily rate, which included costs for phone credit and recharging their smartphone battery. Both Okada riders and surveyors were reimbursed if they had overnight costs due to travel to distant chiefdoms.

## Results

**Fig 2:**
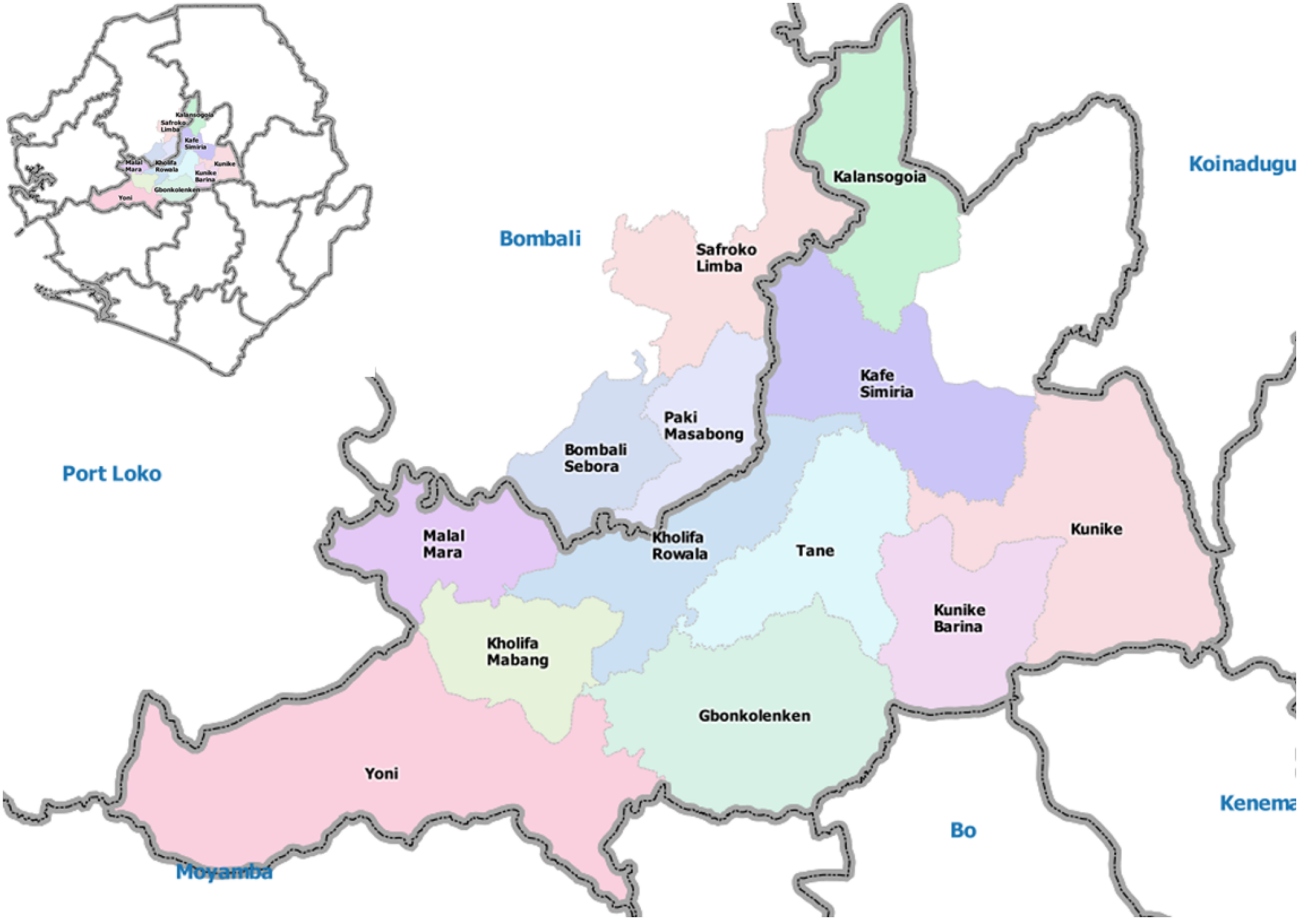
Chiefdoms in Tonkolili and Bombali District in colour represent those surveyed in January 2015. Full country map of Sierra Leone at top left, with coloured areas highlighting the survey area.

Thirteen surveyors were recruited and trained in the use of ODK and OsmAnd software in Magburaka. The survey teams gained sufficient familiarity with the ODK and OsmAnd software within 1–2 hours. During the survey period from 12–31 January 2015, five to ten survey teams worked per day. All completed survey forms were submitted by 1 February 2015. The teams travelled to areas within nine of the 11 chiefdoms in Tonkolili District and three neighbouring chiefdoms in Bombali District (Fig 2).

### Descriptive analysis of survey data collected

Each team surveyed 8–10 villages per day, collecting data from 809 villages in Tonkolili District and 82 villages in Bombali District (Table 1). Along with the official name, 219 (24%) villages had an alternate village name recorded (209 in Tonkolili District, 10 in Bombali District). The names of 887 village chiefs were recorded, of which 841 (95%) provided a mobile number. The surveyors collected the names of 836 local health workers or the name of the local responsible person, if the village did not have a health centre. They also collected the mobile phone number for 708 (85%) health care workers or local responsible people (Table 1).

**Table 1:** Descriptive analysis of information collected from 891 villages by surveyors using ODK software, Tonkolili and Bombali District, January 2015.

District	Chiefdom	Villages surveyed	Villages with alternate name(s)	Village chiefs interviewed	Village chief contact details	Households per chiefdom	Population estimation	Healthcare worker name(s) collected	Healthcare worker contact details	Nearest healthcare facility name
		N	n(%)	n(%)	n(%)	n	n	n(%)	n(%)	n(%)
Bombali	Paki Masabong	52	7 (13)	52 (100)	51 (98)	3,350	19,765	51 (98)	44 (85)	52 (100)
	Safroko Limba^a^	3	0 (0)	3 (100)	3 (100)	233	1,375	3 (100)	3 (100)	3 (100)
	Bombali Sebora	27	3 (11)	27 (100)	26 (96)	10,934	64,511	26 (96)	17 (63)	27 (100)
Tonkolili	Gbonkolenken	179	67 (37)	179 (100)	170 (95)	18,958	111,852	173 (97)	142 (79)	179 (100)
	Kafe Simiria	38	6 (16)	37 (97)	34 (89)	7,932	46,799	32 (84)	24 (63)	38 (100)
	Kalansogoia	57	11 (19)	57 (100)	42 (74)	10,872	64,145	49 (86)	37 (65)	57 (100)
	Kholifa Mabang	55	6 (11)	55 (100)	55 (100)	8,168	48,191	54 (98)	48 (87)	55 (100)
	Kholifa Rowala	101	33 (33)	92 (100)	92 (100)	11,037	65,118	91 (90)	85 (84)	92 (100)
	Kunike^a^	2	0 (0)	2 (100)	2 (100)	60	354	2 (100)	2 (100)	2 (100)
	Kunike Barina	53	10 (19)	53 (100)	50 (94)	7,806	46,055	43 (81)	41 (77)	53 (100)
	Malal Mara	27	4 (15)	27 (100)	25 (93)	5,270	31,093	27 (100)	25 (93)	27 (100)
	Sambaya^b^	-	-	-	-	-	-	-	-	-
	Tane	72	26 (36)	72 (100)	67 (93)	7,131	42,073	71 (99)	59 (82)	70 (97)
	Yoni	232	46 (20)	231 (99)	224 (96)	35,270	208,093	214 (92)	181 (78)	230 (99)

^a^Safroko Limba and Kunike were not extensively surveyed as there were no Ebola cases or people sought care outside of the MSF EMC catchment area.

^b^Sambaya chiefdom was inaccessible during the survey period as the paramount chief issued a lockdown due to EVD fears in January 2015.

As additional variables were added to the survey in the middle of the data collection process, a complete set of data is not available for all items (Table 2). However, of the 27 data variables consistently collected throughout the survey, only 0–1.7% of values were missing (Table 2).

**Table 2:** Information collected from 891 villages by surveyors using ODK software, Tonkolili and Bombali District, January 2015.

Information collected	Data records collected (n)	Missing values (%)
Survey start time	887	0.5
survey end time	887	0.5
Device identification number	891	0.0
Location number	889	0.2
Location latitude	891	0.0
Location longitude	889	0.2
Location altitude	889	0.2
Location precision	891	0.0
Village name	891	0.0
Alternate village name	876	1.7
Village chief name	891	0.0
Village chief phone number	891	0.0
Health worker name	891	0.0
Health worker phone number	891	0.0
Number of village households	891	0.0
Village population*	186	79.1
District	891	0.0
Chiefdom	891	0.0
Section*	185	79.2
Ward	891	0.0
Constituency	891	0.0
Zone*	185	79.2
Has a health centre*	186	79.1
Type of health centre*	185	79.2
Other type of health centre*	185	79.2
Name of nearest health centre	891	0.0
Type of nearest health centre	891	0.0
Village school*	185	79.2
Internal unique name of form	887	0.5
Unique identifier for each form filled	887	0.5
Submission survey time	887	0.5

* Variables added mid-way through the survey

### Population estimates

The estimated number of households in the 11 chiefdoms surveyed was 127,021 (Table 1). Applying the value of 5.9, which is the average number of Sierra Leoneans per household [11], the population estimate within the 891 villages surveyed was 749,424 inhabitants; 663,774 in the nine chiefdoms surveyed in Tonkolili District and 85,650 in the two chiefdoms surveyed in Bombali District (Table 1).

### Cost of the survey

The Okada drivers were paid €25 (SLL 120,000) daily to cover fuel, their daily worker rate and vehicle maintenance. The surveyors were paid €10 (SLL 50,000) daily to cover their daily worker rate, phone credit and smartphone battery recharging. The overall cost for collecting this survey data was €3,395; which equates to €3.80 per village surveyed.

### Mapping validation

**Fig 3:**
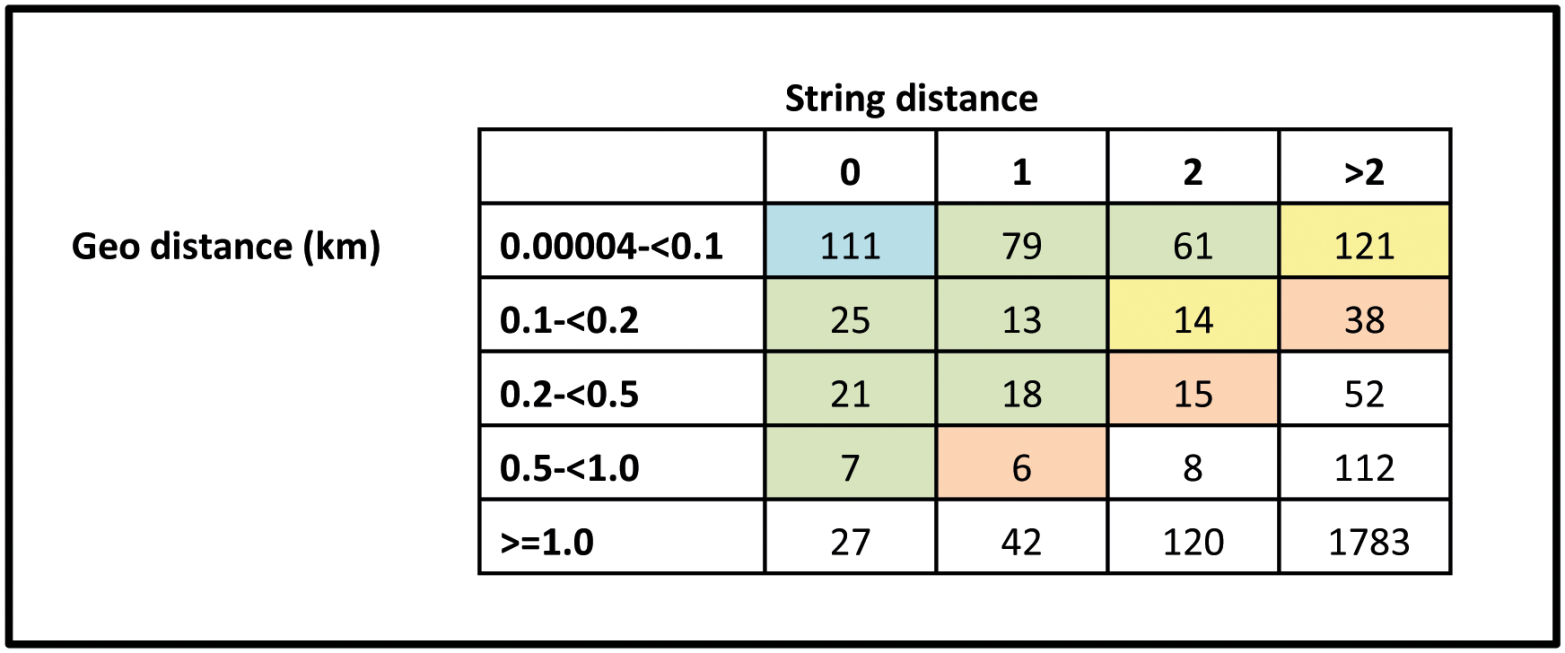
Grid visualization of string and geographical distance combinations for the three top matches per village in the de-duplicated survey list. Note: Due to manual review, the numbers in colored squares do not equate to the precise number considered a match.

In the 11 chiefdoms extensively visited by the survey teams, the pre-existing 2004 Statistics Sierra Leone list of locations contained 1,275 villages. After checking for internal duplicates, the pre-existing list was reduced to 1,272 villages. The survey list contained 970 villages, which were reduced to 891 villages following de-duplication as some teams had unknowingly visited the same villages. Matching of the cleaned pre-existing list against the cleaned survey list found 398 villages considered as duplicates (44% of survey villages), and another 117 villages (13% of survey villages) considered as possible duplicates. Excluding the 398 duplicates, an additional 501 villages identified by the survey team did not meet the matching algorithm criteria. From the pre-existing list, a match on the survey list was not found for 803 villages. Those villages from the pre-existing list were either no longer occupied or had not been visited by survey teams due to EVD travel restrictions or were not within the catchment area of the MSF Magburaka EMC. Combining the pre-existing list and the non-duplicate villages in the survey list thus resulted in a list of 1,773 villages: 398 (22%) in both lists, 874 (49%) only in the pre-existing list and 501 (28%) only in the survey list (Fig 3).

### Use of survey data

Maps using the collected data were created for the MSF Magburaka EMC outreach teams to facilitate hygiene-kit distribution in recently quarantined villages, investigation of suspect EVD cases and for realistic planning of how to get to and from the MSF Magburaka EMC to local villages. EMC data of 139 EVD survivors from Tonkolili District were linked with the survey data, which enabled accurately locating survivors to facilitate follow-up and medical support. The mapping data were shared with the MoHS, Sierra Leone District and National Ebola Response Centres, OpenStreetMap and the MSF GIS team (MSF-OCG). The data were also used as part of the MSF plan to strengthen the Integrated Disease Surveillance and Response system (IDSR) in Tonkolili District.

## Discussion

This project is an example of an innovative and rapid approach to obtain vital mapping and village population data using accessible technology in genuine partnership with the local community in the context of emergency response. The time required to train the surveyors was minimal and no device training was necessary, as all Android smartphones were self-owned. The pairing of local Okada drivers and surveyors allowed rapid data collection and helped overcome possible language barriers. The survey was conducted in less than two weeks, and the geographic information from 891 villages was applied to create up-to-date maps of Tonkolili District.

Following completion of this project in February 2015, the number of EVD cases in Tonkolili district was decreasing [12]. However, at that time, it was unclear if the EVD outbreak would continue to decline. The MSF Magburaka EMC was operational until May 2015, and MSF has continued healthcare activities in the district (MSF, personal communication).

The geolocation data collected was shared with the MSF GIS team (MSF-OCG) [13], who made improved maps for the MSF Magburaka EMC team. Having up-to-date maps enabled rapid identification of villages of recent cases, facilitated hygiene-kit distribution in recently quarantined villages, and aided other MSF activities such as follow-up of EVD survivors. Having the alternate village name was useful for confirming location when the alternate village name was used, which was important given that almost a quarter of villages surveyed had an alternate name. In addition, having the contact information of local leaders and healthcare professionals facilitated giving advance notice of a visit to a village or healthcare facility. Voluntary provision of this information was high during the survey.

We shared the geolocation data with the District and National Ebola Response Centres. We also submitted our data to OSM, who updated their mapping database with the collected geolocation and village name information, thus providing a freely available updated map of Tonkolili District. This survey contributed to improved maps of EVD affected areas produced by the Missing Maps project (http://www.missingmaps.org/), a collaboration involving MSF, the American and British Red Cross, the Humanitarian OSM Team and volunteers working to create free, digital maps for countries affected by Ebola [14]. In 2016, the survey healthcare and geolocation data were used by MSF in collaboration with the MoHS and partners in Tonkolili District to revitalize the IDSR system [15].

We do not know the accuracy of the pre-existing Statistics Sierra Leone list of villages and GPS coordinates we used for validating the survey data collected, although only three village duplicates were identified within the list (0.2% of 1275 villages). It is also unknown how many villages might move, disappear or appear per year in Tonkolili District. This may be dependent on numerous factors, particularly as Tonkolili is a mining area [16]. However, starting with a pre-existing list and updating it would have prevented duplication and allowed a systematic process of visiting each existing village only once, but still using local knowledge and logic to assess villages that did not exist or were missed from the original list. Nonetheless, the villages found in both lists were not wasted effort, as the surveyors obtained substantial extra village information useful for outbreak response.

The advantage of this survey was the minimal set of requirements necessary to securely collect data: one laptop; a server, such as a small mobile device which can operate via internet and range in cost from €10–25; a local web-based aggregation platform; ODK, OsmAnd and QGIS^®^open source software; Android smartphones and affordable local transport. The open-source software used in this survey is known to be virus-free, does not have any advertisement or need for payment, and the collected data belongs to MSF and is secured on an MSF password protected server. In 2014, over half (55%) of Sierra Leonean households surveyed owned a mobile phone [11], therefore using smartphones was feasible by selecting local residents who owned one, and were willing to participate in the study. Thus, there was no need to purchase devices for short term use, and no device training was required. Also, the flexibility of Okada drivers to travel to areas with difficult terrain and their connection with local community enabled us to gain deep knowledge of areas, as well as reach remote and unmapped villages.

A community forms a collective identity with an understanding of local cultural, social, and behavioural backgrounds. This can be difficult for an outsider to immediately recognise and is why MSF programmes are reliant on the involvement of local communities [17). When there was initial difficulty in identifying villages in Tonkolili District, response teams relied on local residents to direct them to the correct locations. This highlighted the importance of local community involvement to inform the implementation and execution of this project and allowed for rapid implementation of the survey, as it was in their home surroundings, local languages, and their own smartphones were used for collecting data. Tonkolili and Bombali districts were stable in January 2015 [18] and collection of data by the surveyors was widely accepted in the communities visited, as everyone recognised their involvement in an important project that contributed to their own community. For the surveyors, it was also an opportunity for work experience in data collection and financial gain.

Our study had several limitations which can serve as lessons learned for future use of this method. First, more knowledge from Okada drivers, locals and satellite imagery could have been used in the final validation of village names. However, this mapping was an emergency operation to meet Ebola response needs for improved mapping information. The data were collected within 11 days under strict curfews and travel restrictions. For future surveys, where possible, more local involvement would be advantageous. However, in countries where safety is an issue, having the option to validate collected data using an algorithm, as we described, would be advantageous. Second, additional variables for data collection were added to the survey mid-way through the project after the potential to collect data rapidly became more evident. As a result, the full set of data items were not collected in villages visited before these additions. However, the key aim was to collect data for mapping, which was collected in full, apart from some chiefdoms and areas within chiefdoms that were off limits to the survey teams due to EVD travel restrictions. In future, it would be advisable to pilot the questionnaire and make the necessary changes before commencing data collection. Third, the population estimation obtained from village chiefs for the 891 villages surveyed was 663,774 in the nine chiefdoms (Gbonkolenken, Kafe Simiria, Kalansogoia, Kholifa Mabang, Kholifa Rowala, Kunike Barina, Malal Mara, Tane, and Yoni) surveyed in Tonkolili District and 85,650 in the two chiefdoms (Paki Masabong and Bombali Sebora) surveyed in Bombali District. These estimates are substantially more than the 2004 census data (280,760 in Tonkolili District; 39,693 in Bombali District) and an overestimation when compared to preliminary results of the census undertaken later in 2015 (435,027 in Tonkolili District; 56,293 in Bombali District) [3, 19]. At the time of surveying, the Sierra Leonean Army were responsible for enforcing quarantines, curfews and travel restrictions throughout the country. Also, in villages and some urban areas, quarantines were imposed, which required that outsiders had to report to chiefs [18]. These abnormal circumstances may have resulted in inaccuracies of village chiefs’ estimates of household numbers. In addition, the average household size we used for Sierra Leone may not have applied to all areas we surveyed. However, given that the survey was developed opportunistically to address an urgent need in the context of an emergency setting, it was still worthwhile trying to estimate village populations. Notably, when estimating populations, it is essential that knowledge about local traditions, social networks and migration patterns are taken into account [20]. Fourth, the matching algorithm for validating the mapping data included only the top three possible matches based on the combination of geographic distance and string distance in the village name. Thus, it is possible that the automated matching process missed some duplicates, particularly if there was substantial string distance between names even though they were actually the same village. Detection of phonetic matches was manual and therefore analyst dependent, so prone to human error. Finally, we did not calculate the village diameter and true range of distance for close, but distinct villages. In addition, it would have been advisable to consider weighting the matching to favour geographical distance over string, and to carry out verification of village distance or the number of households per village by detailed inspection by satellite imagery and/or using local knowledge.

The application of technology has the potential to transform communicable disease control [21]. During the EVD outbreak, advances were made in the use of mobile phones and GPS to support outbreak response activities. In Nigeria, ODK and Form Hub technology were used alongside mapping tools for strategic planning and for the daily follow-up of EVD contacts, identification of cases, case investigation and management [22]. Through the use of this technology, improvements in the reporting were observed. Mobile phones were used in Guinea for intensified EVD surveillance [23], and for community Ebola syndromic surveillance in southern Sierra Leone [24]. Human migration models were built using mobile phone data to help predict patterns of EVD infection [25, 26].

In order to adopt these technologies, their potential needs to be demonstrated through field implementation [21]. Integration of technologies, as described in this article, has the potential to improve response for outbreak surveillance and control. By conducting surveys using accessible technology, necessary information can be collected in a rapid manner that does not require device training.

## Conclusion

Through partnership with local people and using accessible technology, this project rapidly implemented a survey to collect geographic and population data, resulting in updated maps for improved EVD response. These methods could be used and up-scaled at a low cost in areas prone to natural disasters, conflict or epidemic diseases, to support rapid response measures.

## Supporting information

S1 Fig Screenshots of the ODK application and the how to: (1) Select a blank form from the ODK Android application. (2) Select the survey you want to conduct. (3) Select to begin a new survey. (4)-(6) Prompts from ODK survey to collect relevant survey information.
(TIF)

S2 Fig Screenshots of the OsmAnd application and the how to: (1) Record location coordinates. (2) Record favourite locations. (3) Record trail or path taken to locations.
(TIF)

S1 Text How-to guide for use of open-source software to collect mapping and survey data using smartphones. (DOCX)
