# Supplementary figures and images for: Improving mapping for Ebola response through mobilising a local community with self-owned smartphones: Tonkolili District, Sierra Leone, January 2015
Source: PLoS One. 2018 Jan 3;13(1):e0189959. doi: 10.1371/journal.pone.0189959 (PMC5752033; doi:10.1371/journal.pone.0189959)

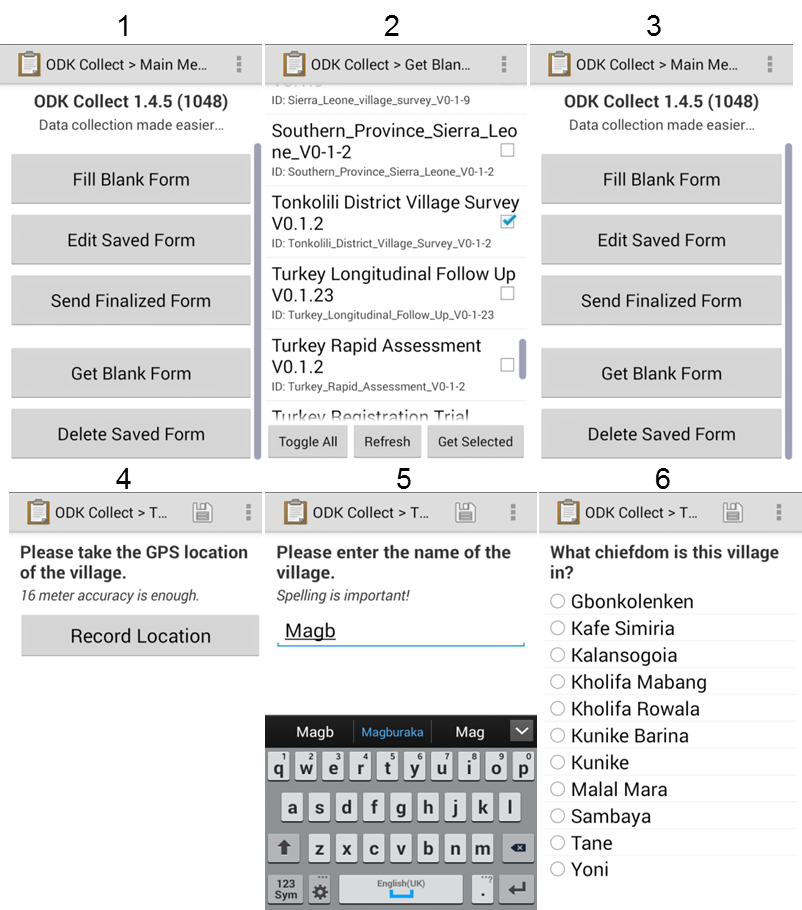

Supplement: S1 Fig — Screenshots of the ODK application and the how to: (1) Select a blank form from the ODK Android application. (2) Select the survey you want to conduct. (3) Select to begin a new survey. (4)-(6) Prompts from ODK survey to collect relevant survey information. (TIF) [file pone.0189959.s001.tif]

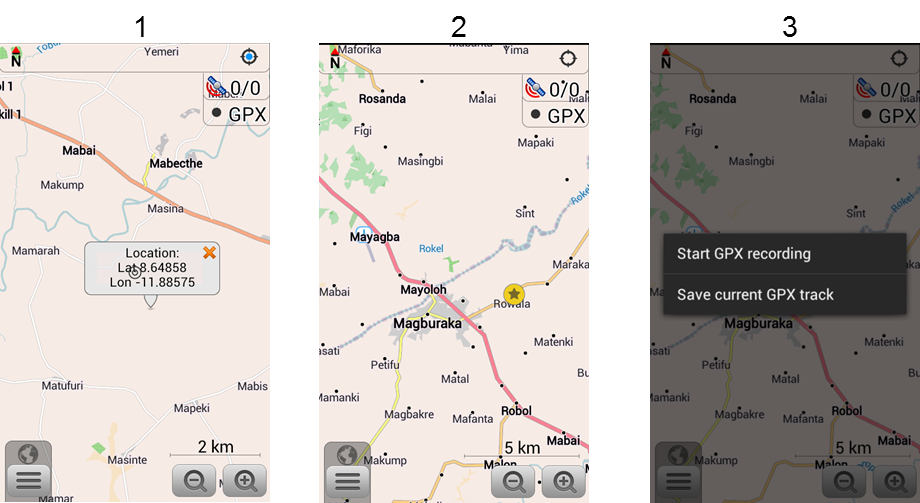

Supplement: S2 Fig — Screenshots of the OsmAnd application and the how to: (1) Record location coordinates. (2) Record favourite locations. (3) Record trail or path taken to locations. (TIF) [file pone.0189959.s002.tif]
