# Supplementary material for: Improving mapping for Ebola response through mobilising a local community with self-owned smartphones: Tonkolili District, Sierra Leone, January 2015
Source: PLoS One. 2018 Jan 3;13(1):e0189959. doi: 10.1371/journal.pone.0189959 (PMC5752033; doi:10.1371/journal.pone.0189959)
Supplement: S1 Text — (DOCX) [file pone.0189959.s003.docx]

**Supporting information**

**How-to guide for use of open-source software to collect mapping and survey data using smartphones**

The finished survey design was uploaded to the password protected MSF web-based aggregation platform by opening a web browser, logging into the aggregation platform, opting to upload a new survey and choosing the Excel file with finalised survey design, then selecting "publish". Android smartphones connected to the aggregation platform then downloaded the survey by selecting the name of the required survey and pressing the "Get blank forms" button in ODK (Supplementary Fig S1). The surveyors collected the survey information by following the prompts in the ODK survey. At the end of each survey day, every surveyor saved the form and exited. When the teams returned to the project base and connected with WiFi, their finished work was uploaded by pressing "Send Finalized Form" in ODK.

S1 Fig: Screenshots of the ODK application and the how to: (1) Select a blank form from the ODK Android application. (2) Select the survey you want to conduct. (3) Select to begin a new survey. (4)-(6) Prompts from ODK survey to collect relevant survey information.

A range of features in OsmAnd were used for this survey including *find your own position,* *favourite point markers* and a recording plugin (Supplementary Fig S2). The *find your own position* feature enabled by GPS or network positioning, caused the map to zoom to the current location by pressing the target button, and allowed the geographic coordinates to be recorded. The *favourites point markers* was used by tapping the screen and selecting ‘*add location to favourites*’, with favourites then available on the OsmAnd dashboard and in the search function. The recording plugin allowed the recording of movement using the phone's GPS or network positioning, which could be used to track the route taken and measure the distance travelled (Supplementary Fig S2).

S2 Fig: Screenshots of the OsmAnd application and the how to: (1) Record location coordinates. (2) Record favourite locations. (3) Record trail or path taken to locations.

After all the surveys were uploaded the number of submissions could be reviewed by logging into the aggregation platform and navigating to the survey. The data were also directly downloaded and reviewed in a Microsoft Excel spreadsheet (.xls) and viewed in a Google Earth map (.kml).
